# An Analytical Method Based on Electrochemical Sensor for the Assessment of Insect Infestation in Flour

**DOI:** 10.3390/bios11090325

**Published:** 2021-09-09

**Authors:** Li Fu, Jiangwei Zhu, Hassan Karimi-Maleh

**Affiliations:** 1 Key Laboratory of Novel Materials for Sensor of Zhejiang Province, College of Materials and Environmental Engineering, Hangzhou Dianzi University, Hangzhou 310018, China; 2 Co-Innovation Center for Sustainable Forestry in Southern China, Nanjing Forestry University, Nanjing 210037, China; jwzhu@nifu.edu.cn; 3 School of Resources and Environment, University of Electronic Science and Technology of China, Xiyuan Ave, Chengdu 611731, China; hassan@uestc.edu.cn; 4 Department of Chemical Engineering, Quchan University of Technology, Quchan 9477177870, Iran; 5 Department of Chemical Sciences, University of Johannesburg, Doornfontein Campus, P.O. Box 17011, Johannesburg 2028, South Africa

**Keywords:** insect infestation, flour, electrochemical sensor, graphene, PEDOT, uric acid

## Abstract

Uric acid is an important indicator of the insect infestation assessment in flour. In this work, we propose a method for uric acid detection based on voltammetry. This technique is particularly considered for the physicochemical properties of flour and contains a simple pretreatment process to rapidly achieve extraction and adsorption of uric acid in flour. To achieve specific recognition of uric acid, graphene and poly(3,4-ethylenedioxythiophene) (PEDOT) were used for the adsorption and concentration of uric acid in flour. The adsorbed mixture was immobilized on the surface of a screen-printed electrode for highly sensitive detection of the uric acid. The results showed that electrocatalytic oxidation of uric acid could be achieved after adsorption by graphene and PEDOT. This electrocatalytic reaction allows its oxidation peak to be distinguished from those of other substances that commonly possess electrochemical activity. This voltammetry-based detection method is a portable and disposable analytical method. Because it is simple to operate, requires no professional training, and is inexpensive, it is a field analysis method that can be promoted.

## 1. Introduction

Wheat is one of the most important food crops, accounting for 22% of total grain production. Wheat flour milled from wheat produces a wide variety of foods and is one of the main sources of nutrition and energy for human consumption. However, a large percentage of flour is lost each year due to insect infestation [1,2,3]. For example, insect infestation causes mold and quality deterioration of flour, which can reduce the edible quality and nutritional value of flour. The secretions and excretions of some pests also have a heavy odor, which directly affects the sensory quality of the flour. Sometimes, grain and food contaminated by pests can cause diseases in humans and animals [4,5,6]. There are two sources of insect infestation in flour. One type comes from the original grain: some moth-eating pests hidden inside the grain. The pests lay their eggs inside the grain prior to harvesting. There are also some parasitic larvae and pupae that live inside the grain. These cannot be removed by normal cleaning and processing techniques [7,8]. Another type of insect infestation source is the pests infected during transportation and storage. Without protection during the distribution of grain, pests can infect grain from the outside environment [9]. Heat treatment of grains is the most commonly used method to stop the deterioration of further insect infections. Research in this area over the last two decades has focused on (1) determining the heat-tolerant stage of a species based on the sensitivity of specific stages at different constant elevated temperatures [10], (2) investigating differences in the sensitivity of stored product insect species to elevated temperatures [11], (3) the degree and duration of insect suppression obtained after heat treatment interventions [12], and (4) investigating thermal mortality kinetic models to predict the survival of heat-tolerant stages [13].

There are many methods to detect insect infestation in grain, but fewer methods can be applied to detect insect infestation in flour. Commonly used methods include flotation, near-infrared spectroscopy, staining, and DNA fingerprinting. The flotation method mainly detects pest fragments in flour [14], and the content of pest fragments in flour has different regulations in different countries. The U.S. Food and Drug Administration (FDA) requires a maximum of 75 insect fragments per 50 g of flour. The flotation method is easily accepted and applied. However, this method has some shortcomings. For example, it is easy to cause the loss of fragments during the actual operation, thus not reflecting the content of fragments faithfully. Secondly, the flotation method is tedious and takes about 2 h per sample, which is time-consuming. Although chemical stains can be used for the detection of pest eggs in flour, only very few staining methods can be used for the detection of pest eggs in flour [15]. Although chemical stains can be used for the detection of pest eggs in flour. However, it is difficult to identify the insect eggs in the dust of flour because they are small and therefore difficult to operate in practice. The NIR spectroscopy method can be used for insect infestation detection because the absorption characteristics of flour containing pest fragments and flour without pest fragments are different at different NIR wavelengths [16]. However, the sensitivity of the NIR spectroscopy method needs further improvement to be well applied to field detection [17]. DNA fingerprinting is an important method for distinguishing differences between organisms of the same species and between organisms of different species from a molecular level. By designing primers specific for the segment of genes that determine the prolongation factor of pest growth and development, followed by a series of molecular marker techniques based on PCR, smaller fragments of pests in flour that are not easily observed with the naked eye can be detected [18,19]. However, there are more bands in DNA fingerprinting and the strength of each band varies, making it more difficult to determine the alleles. Moreover, the cost of this technique is high, which makes it difficult to be widely used at this stage. According to the above, different techniques have their own shortcomings. Therefore, it is of great practical value to develop an efficient, economical, and accurate detection method.

Uric acid is the main end product of purine metabolism in insects and is a trioxypurine with a weakly acidic alcoholic form. The synthesis of uric acid is an important part of the study of insect metabolic pathways [20,21]. Insect secretions are often excreted as uric acid in order to prevent significant loss of water in the body. As an end product of insect nitrogen metabolism, uric acid has been suggested as an indicator of insect contamination on seeds and grain products [22,23]. The current methods for uric acid determination mainly include high performance liquid chromatography, the uric acid enzyme method, and the phosphotungstic acid reduction method [24]. The first two of these methods are specific and sensitive, but their use is limited by the need for special equipment with high cost. Although the phosphotungstic acid reduction method is non-specific, it is widely used because of the simplicity of the required conditions. The electrochemical detection of uric acid has been extensively studied, mainly for medical purposes [25,26,27]. These electrochemical sensors provide highly sensitive detection of uric acid. However, these sensors are designed primarily for the biological environment of the human body and therefore cannot be used directly for the detection of uric acid in flour. In this work, we designed a novel electrochemical sensing detection technique based on the physicochemical properties of flour. This technique allows highly sensitive detection of uric acid in flour. By the level of uric acid, we can further evaluate the insect infestation level of flour. We have performed a comparative corroboration with phosphotungstic acid reduction method and found that this voltammetric detection-based technique possesses good reproducibility, short operation time, and low price.

## 2. Materials and Methods

### 2.1. Materials

Graphene dispersion (2 mg/mL) was purchased from Jiangsu XFNANO Materials Tech Co., Ltd. (Nanjing, China). 3,4-Ethylenedioxythiophene (EDOT) was purchased from Shanghai Guchen Biotech Co., Ltd. (Shanghai, China). Phosphotungstic acid, sodium tungstate, sodium carbonate, uric acid, and ascorbic acid were purchased from Alading Co., Ltd. All of the solutions were made from analytical grade reagents and ultra-pure water. A commercial screen-printed electrode (SPE, Nanjing Youyun Technology Co., Ltd. (Nanjing, China)) with a three-electrode system was used for voltammetry recording.

### 2.2. Preparation of Graphene/PEDOT Composite

The graphene/PEDOT composite was synthesized by a microwave based method [28]. Specifically, 5 mL of graphene dispersion (0.5 mg/mL) was mixed with 1 mL of EDOT and then stirred for 1 h. The mixture was then transferred into a reaction tube and placed into a microwave oven and reacted at 200 W for 5 min. Then, the formed graphene/PEDOT composite was collected after filtration and the washing process.

### 2.3. Electrochemical Sensor Fabrication and Measurement

The synthesized graphene/PEDOT composite was first dispersed into ethanol to form a 0.1 mg/mL solution. Then, 0.2 mL of graphene/PEDOT composite solution was added to 1 mL of uric acid solution or flour extract. The mixed solution was sonicated for 3 min to allow the graphene/PEDOT composite to adsorb uric acid. Subsequently, 5 μL of the above solution was drop coated on the surface of the SPE and dried at room temperature. Cyclic voltammetry (CV) and differential pulse voltammetry (DPV) have been used for measuring the electro-oxidation of uric acid. The electrolyte is a 0.1 M phosphate buffer solution (PBS).

### 2.4. Phosphotungstic Acid Reduction for Uric Acid Measurement

Add 0.5 mL of uric acid solution or flour extract to 4.5 mL of tungstic acid reagent, mix well and let stand for 5 min, then centrifuge at 3000 r/min for 5 min. Add 0.5 mL of 100 g/L sodium carbonate solution to the above centrifuged supernatant, mix well, and let it stand at room temperature for 10 min. The absorbance was measured by UV spectrophotometer at 660 nm.

### 2.5. Real Sample Preparation

*Tribolium castaneum* with a clean and vigorous body were selected, reared on flour and incubated in a biochemical incubator. After 48 h of incubation, the adult *Tribolium castaneum* were separated from the flour by passing through a 60 mesh grain sieve. The sieved flour was then sieved through an 80 mesh grain standard sieve to separate the eggs (the eggs were produced with mucus, they can adhere to the flour and are able to remain on the 80 mesh grain sieve). The eggs were collected from the sieve and placed in a Petri dish for use. Add flour with different numbers of eggs to the test tubes, then add 2 mL of distilled water, and 1 mL of 0.1% sodium carbonate solution. The suspension was formed after shaking. The flour extract was then transferred to a centrifuge tube and centrifuged at 10,000 r/min for 10 min to obtain the flour extract after the eggs were completely broken.

## 3. Results and Discussion

**Figure 1:**
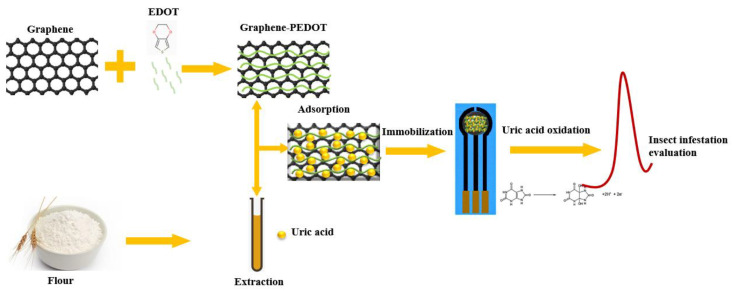
Schematic diagram of the fabrication of our proposed analytical sensor for the assessment of insect infestation in flour.

Figure 1 shows a schematic diagram of the fabrication of our proposed analytical sensor. In this detection method, the detection of uric acid is not simple oxidation on the electrode. The uric acid molecules are first adsorbed and concentrated by the graphene-PEDOT nanocomposite, which is then immobilized on the SPE electrode surface. This process not only improves the sensitivity of the uric acid oxidation current but also helps to improve the interference resistance in the actual sample detection. Graphene can be used to adsorb uric acid molecules due to its high specific surface area [29]. At the same time, graphene has very good electrical properties and can improve the electrochemical signal [30]. PEDOT acts as a catalyst in composite materials. Previous studies demonstrated that uric acid can be electrocatalytically oxidized on the surface of PEDOT-modified electrodes [31]. This electrocatalytic behavior allows the sensor to perform detection of uric acid avoiding the influence of other common interferents, since the amount of uric acid and the degree of insect infestation show a positive relationship. The oxidation current of uric acid recorded in this assay can be used to evaluate insect infestation in flour.

**Figure 2:**
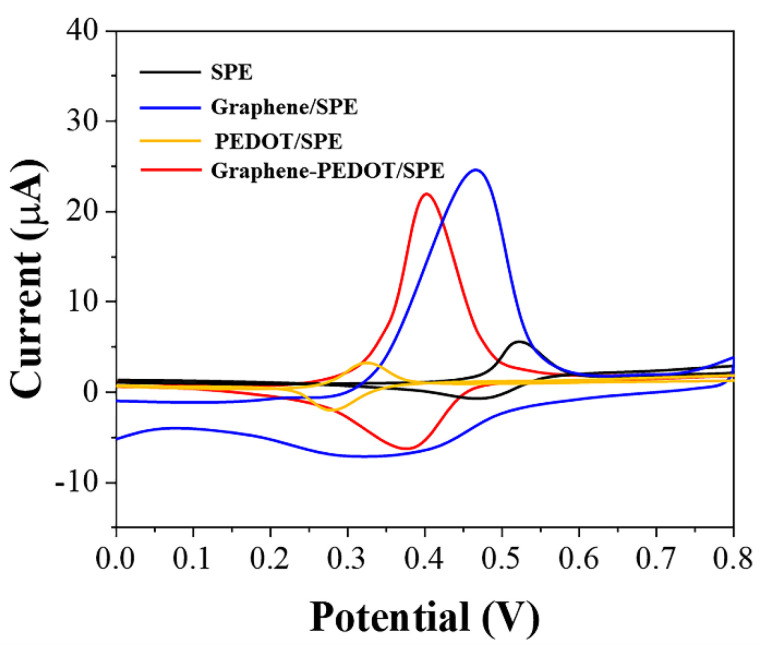
CVs of SPE, PEDOT/SPE, graphene/SPE, and graphene−PEDOT/SPE towards 10 μM uric acid.

The electrochemical oxidation behaviors of uric acid at different electrodes were shown in Figure 2. It can be seen that uric acid can be oxidized directly on the SPE with a very easily recognizable oxidation peak at 0.62 V, corresponding to a current of 4.7 μA. The small current obtained in this case was not only due to the limitations of the detection performance of the SPE itself; only a fraction of the free uric acid molecules were instinctively involved in the electrochemical reaction. If the uric acid molecule and PEDOT were co-mixed and then immobilized on SPE, the oxidation potential of uric acid shifts negatively. However, the current value is lower than the direct oxidation of uric acid on SPE. It can be seen that, although the presence of PEDOT can achieve an electrocatalytic effect on the oxidation of uric acid, the poor electrical conductivity of PEDOT leads to a decrease in the efficiency of electron transfer. In contrast, graphene adsorption of uric acid can greatly increase the current of the oxidation peak. In addition, graphene adsorption can allow a small negative shift of the oxidation potential of uric acid. This catalytic property is caused by the defects on the surface of graphene and functional groups on the boundary, which is consistent with our previous report [32]. The best results were observed on SPEs of graphene-PEDOT composites adsorbed with uric acid. The oxidation of uric acid on this electrode not only has a very significant negative potential shift but also possesses a very high current response. Therefore, we used graphene-PEDOT to first adsorb and concentrate uric acid to improve the sensitivity of the sensor.

**Figure 3:**
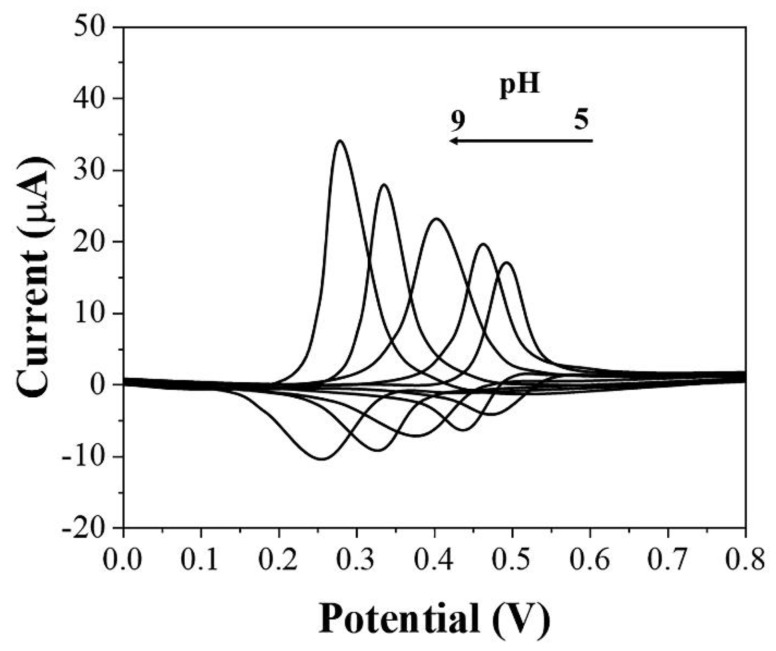
CVs of graphene−PEDOT/SPE towards 10 μM uric acid under different pH conditions of PBS.

Electrolytes can influence the electrochemical behavior of uric acid at the electrode. In this work, we investigated the electrochemical response of uric acid after adsorption by graphene-PEDOT in PBS under different pH. As shown in Figure 3, there is a positive correlation between the intensity of the oxidation current of uric acid and pH. As the pH increases, the current of the oxidation peak becomes larger. In addition, the oxidation potential of uric acid was influenced by pH as well. As the pH increased, the oxidation potential of uric acid also underwent a negative shift due to the involvement of protons in the electrochemical reaction [33]. Although a lower potential for electrochemical sensing helps detection, we finally chose pH 7 as the electrolyte environment for sensor operation considering the oxidation potential overlapping of some other substances, such as ascorbic acid [34].

**Figure 4:**
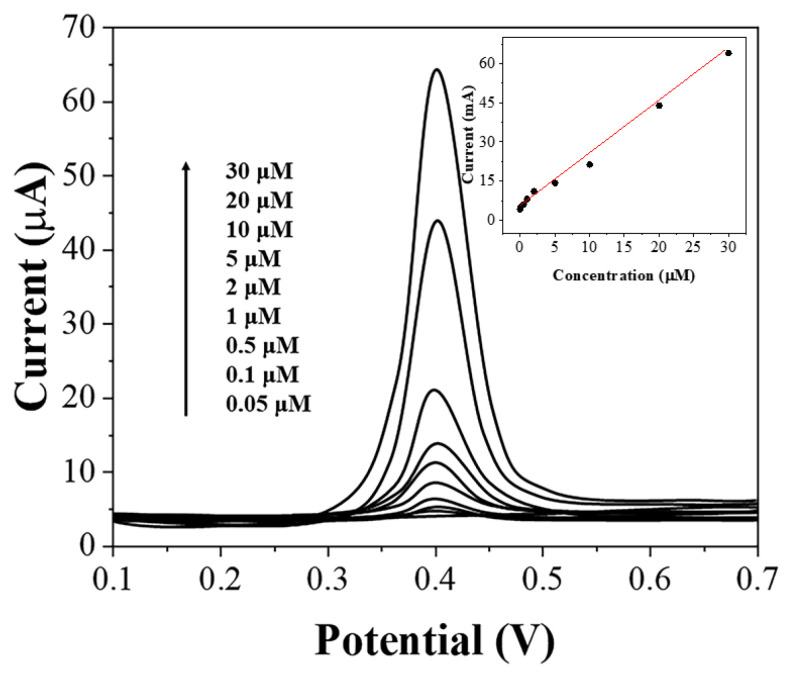
LSV of graphene-PEDOT/SPE towards 0.05–30 μM uric acid in PBS (pH = 7).

Before performing the detection of uric acid in flour, we verified the detection performance of the assembled sensor. Figure 4 shows the linear sweep voltammetry (LSV) of graphene-PEDOT adsorbed with different concentrations of uric acid and then immobilized on SPE. As expected, the oxidation current of uric acid increases linearly with an increasing concentration of uric acid in the range of 0.05–30 μM. The detection limit can be calculated as 15 nM based on S/N = 3. Table 1 compares the effectiveness of the proposed sensor with other reported literature. It can be seen that our sensor is particularly sensitive at lower concentrations but has no advantage at higher concentrations of uric acid. Since our sensor is used for the detection of uric acid in flour, it needs to be sensitive to lower concentrations of uric acid. This is why we need to use graphene-PEDOT for adsorption and concentration of uric acid molecules. We tested five prepared sensors and they showed excellent performance for the oxidation of 10 μM uric acid. The RSD of the current value was only 3.2%. The selectivity of the sensor was then tested. Common small molecules, such as 10-fold of ascorbic acid, fructose, and sucrose do not affect the test results.

**Figure 5:**
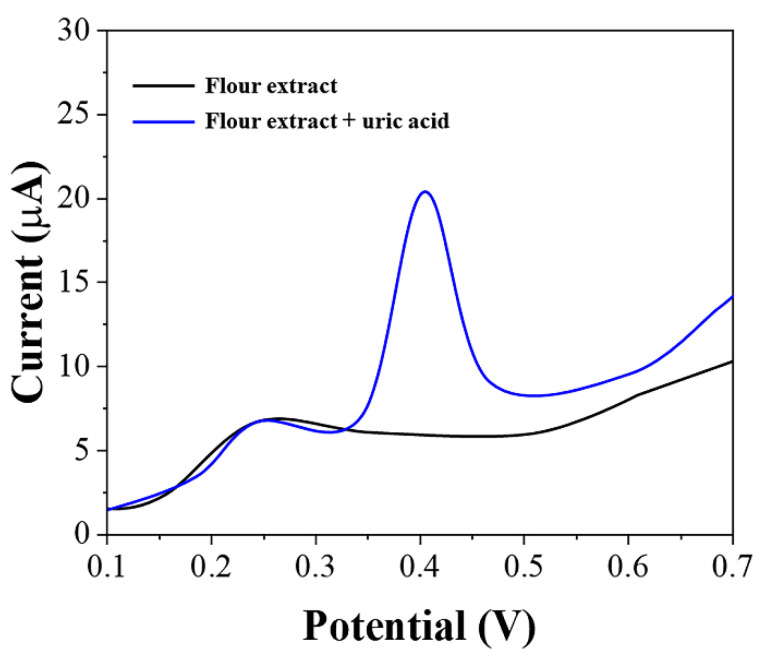
LSV of graphene-PEDOT/SPE towards flour extract and flour extract with the addition of 10 μM uric acid.

The extract of the flour must contain other substances possessing electrochemical activity. Figure 5 shows the LSV of graphene-PEDOT after adsorption of the flour extract. From the figure, it can be seen that there are substances in the flour extract that can be electrochemically oxidized. These substances are complex in composition and are mainly small antioxidant molecules in wheat. We then added 10 μM of uric acid to the flour extract and could see that the oxidation peaks of uric acid did not overlap with those of other substances. This is the reason why we selected pH 7 as the detection environment. At other pH conditions, the electrochemical oxidation of these substances would partially overlap with the oxidation peak of uric acid, which in turn would affect our determination of uric acid content.

We then tested the linear detection range of the proposed electrochemical assay for uric acid in flour extracts. Since graphene-PEDOT adsorbs other substances in addition to uric acid molecules during the adsorption process, the detection performance of the sensor is slightly reduced compared to the previous detection of a single uric acid. However, this analytical method still provides a linear detection of 0.1–20 μM uric acid. The detection limit can reach 37 nM. We then tested the proposed sensor using the eggs of *Tribolium castaneum* as an actual insect infestation. Phosphotungstic acid reduction was used as a conventional assay for comparison. Table 2 shows the comparison of the results of the two different detection methods. As can be seen from the table, the number of *Tribolium castaneum* eggs is directly proportional to the amount of uric acid. The results of the electrochemical sensor are very similar to those of the phosphotungstic acid reduction method, proving that our proposed method can be well applied to the determination of uric acid in flour.

## 4. Conclusions

In conclusion, a novel electrochemical sensor was prepared for the determination of uric acid content in flour and further reflected the insect infestation situation. Graphene-PEDOT was first synthesized for the adsorption and concentration of uric acid from flour extracts. This step allows the electrochemical sensor to detect very low concentrations of uric acid. After optimizing the parameters, the proposed electrochemical sensor can provide linear detection of uric acid between 0.05 and 30 μM. In real flour samples, the linear detection range of the sensor is slightly reduced due to interference from other substances. However, the sensor is still able to achieve linear detection in the 0.1–20 μM range.

## Figures and Tables

**Table 1:** Comparison of the proposed sensor with other reported electrochemical sensors.

Sensor	Linear Detection Range	Limit of Detection	Reference
ZnO-Au NPs	4–400 μM	2.375 μM	[35]
CTAB-GO/MWCNT	3–60 μM	1 μM	[36]
GO/TmPO_4_	10–100 μM	5.9 μM	[37]
MoS_2_-PANI/RGO	1–500 μM	0.36 μM	[38]
PDDA-CNT-G/SPE	5–50 μM	4.4 μM	[39]
MWCNTs/MGF/GCE	5–100 μM	0.9 μM	[40]
graphene-PEDOT/SPE	0.05–30 μM	15 nM	This work

**Table 2:** Results and uric acid measurement using the proposed electrochemical sensor and the phosphotungstic acid reduction method.

Egg Number	Phosphotungstic Acid Reduction		Electrochemical Sensor	
	Absorbance	Uric Acid Concentration	Current	Uric Acid Concentration
0	0.425	0.000 μM	4.020 μA	0.000 μM
2	0.442	2.511 μM	9.112 μA	2.504 μM
4	0.449	3.781 μM	11.241 μA	3.779 μM
6	0.455	5.713 μM	14.647 μA	5.710 μM
8	0.462	7.101 μM	19.202 μA	7.095 μM

## Data Availability

Data sharing not applicable.
